# Development of attentional disengagement in typically developing children and children with elevated levels of attentional deficits

**DOI:** 10.1007/s00221-025-07177-7

**Published:** 2025-10-21

**Authors:** Beleke de Zwart, Dirk van Moorselaar, Roy S. Hessels, Nanda Rommelse, Stefan Van der Stigchel

**Affiliations:** Department of Experimental Psychology and Helmholtz Institute, Utrecht University, Heidelberglaan 1, 3584 CS Utrecht, The Netherlands

**Keywords:** YOUth cohort, Gap-Overlap task, Longitudinal, Attention, Development

## Abstract

The ability to flexibly disengage and shift attention allows us to successfully interact with our environment and develops across infancy, childhood and adolescence. The aim of the present study was to investigate how attentional disengagement abilities develop. We leveraged a robust longitudinal dataset of the Gap-Overlap task of the YOUth cohort with over 3500 children tested at one or more timepoints to track the maturation of attentional control across infancy, childhood and adolescence (5 months, 10 months, 3 years, 6 years, 9 years, 12 years). While we replicated established group-level developmental patterns showing progressive improvement in saccadic reaction times, the time between the appearance of the target and the initiation of the saccade, our findings revealed individual variation in developmental trajectories of the gap effect that cannot be reliably predicted from performance at earlier timepoints. Although we found no associations between Gap-Overlap performance and (parent-reported) attention-related behaviors (n = 99–734 depending on age and measures), this may reflect the difficulty of identifying early markers in highly heterogeneous neurodevelopmental disorders and could be limited by examining subclinical traits rather than diagnosed conditions. The increased understanding of individual developmental patterns established here is a prerequisite for the identification of atypical patterns in clinical assessment. Future studies could build on this work by investigating atypical attention profiles across development and their relationship to Gap-Overlap task performance, particularly in clinical populations where attentional differences may be more pronounced.

**Supplementary Information:**

The online version contains supplementary material available at 10.1007/s00221-025-07177-7.

## Introduction

Attention is not a static ability we are born with, but comprises dynamic skills that develop and refine throughout our lifetime. The study of attentional development has been approached through various paradigms, targeting for example selective attention through visual search tasks (e.g., Trick and Enns 1998; Scerif et al. 2004), continuous performance tasks as index of sustained attention (e.g., Lin et al. 1999), or Go/No-Go, Stroop tasks and stop-signal tasks for executive or attentional control (e.g., Cieslik et al. 2015; Durston et al. 2007; Gerstadt et al. 1994). While these paradigms have advanced our understanding of attentional development, many require complex verbal instructions and sophisticated motor responses, limiting their applicability to infants and very young children. However, fundamental attentional processes such as disengagement and shifting can be studied from the earliest stages of development. Between 1 and 3 months, children have difficulty looking away from a stimulus (Atkinson et al. 1992), but the ability to disengage and shift attention becomes increasingly efficient as they grow older. Attentional disengagement and shifting allows us to prioritize relevant information, yet this ability transforms from a limited skill in infancy to a more sophisticated mechanism in later childhood (Hood and Atkinson 1993). A well-established gaze-contingent paradigm for studying specifically visual attentional shifting and disengagement is the Gap-Overlap task (e.g., Saslow 1967). In this task, participants fixate on a central stimulus and then must disengage from the center to orient towards a peripheral target. These attentional disengagement and shifting processes, operationalized by saccadic reaction times (SRTs), are thought to be underlying mechanisms of cognitive control (Mackie et al. 2013; Moyano et al. 2023).

The prosaccade version of the Gap-Overlap task consists of several conditions; in the baseline condition, the disappearance of the central stimulus occurs simultaneously with the appearance of the peripheral stimulus, whereas in the gap condition the central stimulus disappears prior to the onset of the peripheral stimulus, facilitating automatic attentional disengagement (Elsabbagh et al. 2013). In the overlap condition however, active disengagement from the central stimulus is required as the stimulus remains present when the peripheral target emerges. The consistently longer saccadic reaction times in the overlap condition compared to the gap condition, signal slower attentional disengagement when the central stimulus remains present (e.g., Saslow 1967; Reuter-Lorenz et al. 1995; Fischer and Weber 1993).

The developmental literature unequivocally suggests that attentional shifting and disengagement abilities improve across the lifespan (e.g., Hood and Atkinson 1993; Klein and Foerster 2001). Nevertheless, there is some discrepancy about when these abilities start to develop. For example, some studies reporting shorter SRTs at 6 months compared to 12 months in both the gap and overlap condition (Nakagawa and Sukigara 2019), whereas others report a decrease in SRTs already between 6 months and 9 months and 16–18 months (Moyano et al. 2023), 6 months compared to 12 months (Alahyane et al. 2016) or 4 months compared to 8 months (Gredebäck et al. 2006). Even though Moyano et al. (2023) conclude a significant SRT reduction with age, their longitudinal findings also indicate that disengagement abilities as indexed by the overlap condition are less consistent after 9 months compared to the gap condition. Accordingly, this hints towards individual differences in developmental progress (Moyano et al. 2023). While it is valuable to look at these effects across conditions, most of these studies have relatively small sample sizes. Moreover, less is known about inter-individual variation in this attentional maturation process since most studies have relied on summary-level latency effects of cross-sectional data rather than studying within-individual patterns over time. The high powered YOUth cohort offers a unique opportunity to investigate how attentional disengagement and shifting unfolds across the lifespan both between different age groups and within individuals longitudinally (Onland-Moret et al. 2020).

In the current work, we focus on the latencies to shift attention to the peripheral stimulus in the three general Gap-Overlap conditions (Baseline, Gap, Overlap). Above and beyond reporting these summary latency effects, we focus on within-subject difference scores to dissociate between the specific attentional mechanisms while accounting for baseline individual differences in processing speed. Therefore, leveraging this robust and high powered dataset, we seek to further investigate the development of attentional disengagement and shifting over the course of infancy, childhood and early adolescence. Generally speaking, we expect to replicate the observed decrease in saccade latencies with age and will furthermore focus on the gap effect. The gap effect serves as an established psychometric index of attentional disengagement in infants, and the current work aims to improve our understanding of its development across early childhood (Cousijn et al. 2017). Then, the longitudinal design allows us to address the developmental trajectory within the same individuals, thereby being the first to examine whether performance at a younger age is predictive for performance at later time points.

Beyond obtaining a more fine-grained understanding of the typical attentional development, these findings could potentially aid in early identification of children with atypical trajectories. Attentional dysfunctions are a clinical hallmark of several neurodevelopmental disorders, for example Attention-Deficit/Hyperactivity Disorder (ADHD). ADHD is the most commonly diagnosed neurodevelopmental disorder, yet has a very heterogeneous clinical profile (Luo et al. 2019; Nigg et al. 2020).

More specifically, attentional problems in individuals with ADHD might manifest in several ways. Whereas some might struggle to disengage and rapidly shift their attention to novel information, others may have difficulty with staying focused on one object. Better understanding of the diverse phenotypic expression can benefit in early diagnosis and possibly improve tailored interventions. This is especially important as next to the increased risk for other psychiatric disorders and negatively impacting the individuals’ daily life, other societal aspects include, but are not limited to, educational difficulties, healthcare burden and strained social relationships (Luo et al. 2019).

However, evidence remains inconclusive for altered performance in specifically the Gap-Overlap task in children with an established ADHD diagnosis. Some studies report no difference in saccade latencies or altered accuracy (e.g., Bellato et al. 2023; O’Driscoll et al. 2005; Maron et al. 2021; Karatekin and Asarnow 1998; Karatekin 2006; Hanisch et al. 2006), whereas others describe patterns of longer SRTs, greater intra-subject variability and/or premature saccades (e.g., Munoz et al. 2003; Huang and Chan 2020; Klein et al. 2003; Mostofsky et al. 2001). As the YOUth cohort provides next to extensive eye-tracking metrics on the Gap-Overlap task also questionnaires tapping on ADHD-related subscales and symptoms, we aim to exploratively investigate whether underlying ADHD pathology is reflected in altered saccadic performance on the Gap-Overlap task.

In summary, although previous studies have examined attentional disengagement using the Gap-Overlap task, most relied on cross-sectional designs with small samples. Here, we use a large-scale longitudinal dataset to examine (1) how attentional disengagement develops over infancy and childhood, (2) whether early performance predicts later attentional control, and (3) whether attentional disengagement relates to attention-related behaviors.

## Methods

*Participants*. For the present paper, we requested Eye-tracking and questionnaire data from the YOUth cohort. In short, this large accelerated longitudinal study design consists of two cohorts (0–6 years and 9–12 years) with participants recruited in Utrecht and its neighboring communities (Onland-Moret et al. 2020). The YOUth study was approved by the Medical Research Ethics Committee of the University Medical Center Utrecht and all participants’ parents provided written informed consent. A brief overview of the YOUth study is available from https://www.uu.nl/en/research/youth-cohort-study. For a detailed description regarding in- and exclusion criteria, recruitment targets, follow-up procedures and applied measurements (experiments, questionnaires, etc.), we refer the reader to Onland-Moret et al. (2020). The dataset of the study is partially longitudinal, partially cross-sectional, with some children participating at multiple timepoints (either consecutively or nonconsecutively) and some participating only once. In total we obtained eye-tracking data on the Gap-Overlap task of 3531 different children (2241 Baby and Child Cohort, hereafter referred to as Baby Cohort; 1290 Child and Teenage Cohort, hereafter referred to as Teenage Cohort), and questionnaire data of 2003 children for the CBCL and 1013 children for the CBQ. After data cleaning procedures (see Supplementary Table S1 for an extensive overview), 3441 unique children were included in the eye-tracking analyses. See Table 1 for an overview of the cohorts.

**Table 1:** Overview of participants and wave per task

Cohort	Wave		Age (months)	Gender	Obtained datasets gap-overlap task from cohort	Included datasets gap-overlap task	Collected trials (of included participants)		
							48	60	Else
Baby and child	5 months		*M =* 5.42, *SD* = 0.84, [3–8]	829 Girl 805 Boy	1900	1634	302	1146	186 [17–60]
Baby and child	10 months		*M* = 10.48, *SD* = 1.10, [8–18]	808 Girl 825 Boy	1809	1633	663	749	221 [8–84]
Baby and child	3 years		*M* = 43.32, *SD* = 9.88, [23–60]	490 Girl 482 Boy	1009	972	573	315	84 [14–47]
Baby and child	6 years		*M* = 73.62, *SD* = 6.63, [60–91]	181 Girl 143 Boy	336	324	289	33	2 [26–27]
Child and teenager	9 years		*M* = 113.4, *SD* = 10.25, [95–132]	719 Girl 530 Boy	1269	1251	1235	13	3 [30–41]
Child and teenager	12 years		*M* = 158.15, *SD* = 13.01, [133–187]	213 Girl 160 Boy	382	374	374	0	0

Note that our age groups are jittered around the target age (in months). Collected trials refers to the number of trials children completed. The standard procedure for the task included 48 trials. If participants failed to achieve at least 12 valid trials per condition, they received an additional block of 12 trials, totaling of 60 trials. A small number of participants completed different trial numbers (referred to as ‘else’) arguably due to experimental errors or early termination.

*Apparatus*. A Tobii Pro TX300 eye-tracker (Tobii Technology, Stockholm, Sweden) with an integrated 23-inch monitor (1920 by 1080 pixels; 60 Hz refresh rate) or the Tobii Pro Spectrum eye tracker with an integrated 23.8-inch monitor (1920 × 1080 pixels, 60 Hz) was used to record gaze location on screen. The Tobii TX300 ran at 300 Hz and the Spectrum at 600 Hz and communicated through Matlab (MathWorks Inc., USA) via the Tobii SDK. Stimulus presentation was handled through PsychToolbox (Brainard 1997).

*Gap-overlap task*. To obtain insights in the visual attentional shifting abilities at different ages, an adapted version of the prosaccade condition of the Gap-Overlap task was used (Elsabbagh et al. 2013; Van der Stigchel et al. 2017; Cousijn et al. 2017). In this task, participants fixate at a central stimulus and are instructed to look at the appearing peripheral stimulus. Trials started with a clock within 3 × 3 cm (2.8° × 2.8°) looming (max 4.2°) and throbbing (max 4.6°) at the center of the screen to attract the participant’s attention (https://github.com/UtrechtUniversity/youth-tasks-matlab-kkc/blob/master/saccade/saccade_trial.m; https://github.com/UtrechtUniversity/youth-tasks-matlab-kc/blob/master/saccade/saccade_task.m). After the participant fixated the central stimulus, it started spinning with a speed of 500°/s to maintain the participant’s attention. After a variable interstimulus interval (600–700 ms) to decrease anticipatory saccades, a peripheral stimulus (a yellow oval, 2.6° × 2.6°) was presented at approximately 20.8° to the left or right from the central stimulus. Values in degrees are given assuming 62 cm distance to screen (TX300); for the Spectrum it was scaled to maintain similar dimensions on retina (64 cm distance to screen, slightly larger stimuli on screen). The small angle approximation was used.

The task contained a gap, overlap, and baseline condition. Trials were presented in 4 blocks of 12 trials, with condition order randomly determined. In the gap condition, central stimulus offset was 200 ms before peripheral stimulus onset. In the overlap condition, the central and peripheral stimulus remained simultaneously on screen. In the baseline condition, the peripheral stimulus onset was at the same time as central stimulus offset. The peripheral stimulus stayed on screen until the participant fixated it or until 2000 ms elapsed. Upon fixating the peripheral stimulus, or if 2000 ms elapsed, the peripheral stimulus spun or throbbed over 1000 ms. This feedback was combined with various sounds (e.g., a car horn, a bell). Neither speed nor accuracy were stressed. The task consisted of 16 trials per condition with left and right peripheral stimulus onsets counterbalanced. If less than 12 trials per condition were marked as successful by a conservative online gaze contingent check, additional trials were presented. Despite relatively low trial count, which reflects a compromise of working with young children in demanding eye-tracking paradigms, aggregation across many participant reduces the impact of individual measurement error and provides robust statistical power for detecting developmental effects. See Table 1 for an overview per age.

*Procedure*. Participants were positioned at a distance of 65 cm from the TX300 eye-tracker or 67 cm from the Spectrum. Infants ranging between 5 months and 3 years were positioned in a car seat (Hessels and Hooge 2019). For the 6, 9 and 12 years aged, a chinrest was used to ensure the same distance and position throughout the experiment. Hereafter, an operator-controlled calibration was run, which consisted of colored expanding and contracting spirals presented at the four corners and the center of the screen. The spirals were accompanied by a sound. A web-cam was used to monitor the participant. When the operator judged the participant to be looking at the spiral, a button was pressed, after which the colored spiral (red, green, yellow, purple or blue) contracted (between 4.0° and 5.4° at 0.8 Hz following a sinusoidal wave) and was calibrated (Hessels et al. 2015). The operator judged the calibration output from the Tobii SDK, after which a decision was made to accept the calibration or re-calibrate. After the calibration was accepted, the Gap-Overlap task started. Throughout the experiment, the participant was monitored through a live video feed. The task including calibration lasted approximately 10 to 15 min. The antisaccade version of the Gap-Overlap task, in which participants are instructed to look at the exact opposite of where the peripheral target appears, followed the pro-saccade version of the task with a short break and a calibration in between for ages 6 years, 9 years and 12 years. In current paper, we only include the prosaccade condition.

*Preprocessing eye-tracking data*. Eye-tracking data is preprocessed based on fixation classification by means of I2MC v2.0 using default parameters (Hessels et al. 2017). Fixations were assigned to specific Areas of Interest (AOIs), one for the left part of the screen, one for the middle and one for the right. After AOI assignment, SRTs were defined for each trial by subtracting fixation end time (the last fixation moment on the center) from the peripheral stimulus onset time. Hereby, to count as successful trial, fixations in the center AOI needed to be followed by a fixation on the left or right AOI. When multiple instances of a fixation on the center AOI followed by a fixation on the left or right AOI occurred, the first instance was used, the first one for each trial was used.

*Outcome measures*. For all analyses, we report the latencies to shift attention to the peripheral stimulus in the three general Gap-Overlap conditions (Baseline, Gap, Overlap). Above and beyond these task specific measures, we then computed within-subject difference scores to dissociate between the specific attentional mechanisms while accounting for baseline individual differences in processing speed. These are the gap effect (median SRT overlap - median SRT gap) as index of attentional disengagement and, although not standard reported, the facilitation effect (median SRT baseline - median SRT gap) as index of the efficiency of anticipatory attentional processes.

*(Early) Child behavior questionnaire ((E)CBQ)*. The (E)CBQ is an age-specific questionnaire filled in by the caregiver to measure temperament in young children (Rothbart et al. 2001; Putnam et al. 2014; Onland-Moret et al. 2020). The ECBQ (translated by de Kruif, Willekens, de Schuymer) is appropriate for children between 18 months and 36 months. Therefore, depending on the exact age of the child during the visit at the 3 years timepoint, either the ECBQ or the CBQ was administered. At age 6, the CBQ (translated by M. Majdandžić), suited for children aged 3–7 years, was administered. The ECBQ consists of 39 questions and the CBQ of 87 questions, with answers ranging on a Likert scale from 1 (Never) to 7 (Always). For each subscale, the mean of the according items are calculated and converted to T-scores based on normative data of Rothbart et al. (2001). We report the Attentional Shifting subscale from the ECBQ, consisting of 8 items, which captures the ability to flexibly shift attention between tasks or activities. Additionally, we used the Attentional Focusing subscale from both the ECBQ and CBQ, which reflects the ability to maintain attention on task-related activities (based on 6 items).

*Child behavior checklist (CBCL)*. The Child Behavior Checklist (CBCL) is utilized as index of atypical (problematic) behavior in social interactions, with a version for ages 1.5 till 5 and another for ages 6–18 (Achenbach and Rescorla 2001; for Dutch: Verhulst et al. 1996). The questionnaire comprises of 118 questions for which participants, either parent or child, rate each question on a 3-point scale from *Not at all* (0), to *A little or sometimes* (1), to *Clearly or often* (2). Items of a subscale are summed, with higher scores indicating higher experienced problems. A Total Problems Score can be generated by summing all subscale scores. As current study focuses on attentional deficits, we calculated for each participant the score on the “Attention-Deficit/Hyperactivity Disorder (ADHD) Hyperactive-Impulsive and Inattentive types” subscale. However, it should be noted that a score on this DSM-oriented scale is not equivalent to a DSM-diagnosis. T-scores are obtained based on the summed scores and compared to the norms of a non-reffered sample (depending on age (6–11 vs. 12–18) and gender). T-scores of > 70 are in clinical range, between 65 and 69 in the borderline clinical range and equal or lower to 64 are considered healthy.

## Results

*Preprocessing*. Data was preprocessed in a Python environment using custom scripts. First, we carefully examined the distribution of children’s ages within each wave, as follow-up assessment of children was intentionally jittered around the predefined waves. We removed participants for which there was overlap with the consecutive time bin or when defined as an extreme (n = 1 for 5 months, n = 14 for age 3 years, n = 2 for age 12 years). One participant was removed due to a technical error. Data quality was ensured by removing trials based on precision and data loss (as in Belteki et al. 2025; for a different eye-tracking experiment in the YOUth cohort). Precision was defined as the median root mean square of sample-to-sample deviations in the gaze position signal, calculated within a 0.1-s moving window for each trial. The deviations were calculated separately for the horizontal and vertical coordinates of each eye and combined using Pythagoras’ theorem. Trials with a mean RMS across the two eyes of > 2 degrees were removed. Then, trials were excluded with proportion of data loss (i.e. no gaze coordinates) averaged over 2 eyes > 0.7. This data quality cleaning resulted in 47,928 trial loss (13.92%). Data filtering procedures were performed in which trials were removed where either no eye movements were made or where the eye movement was towards the wrong side of the screen. Consecutively, data was trimmed based on reaction times to exclude trials with premature saccades (< 150 ms) or trials that indicated inattentiveness or (potentially) failed disengagement (> 2000 ms). For each participant, we removed conditions in which less than 4 trials remained. After, we calculated the median SRT per participant per condition. The resulting dataset (n = 183,447 trials, 53.26% of the original number of trials; 2170 unique participants Baby Cohort; 1271 unique participants Teenage Cohort) was used for eye-tracking analysis. Table S1 for an extensive overview of the outlier removal procedure and the percentages of removed trials per age group per data cleaning step and Table S2 for the percentages of removed trials per age group per condition when eye movements were made towards the wrong side. Statistical analyses were performed in Python version and JASP version 0.18.3.0. Raw data can be requested via (https://www.uu.nl/en/research/youth-cohort-study/request-youth-data), the experimental code is available via (https://github.com/UtrechtUniversity/youth-tasks-matlab-kkc/blob/master/saccade/saccade_task.m) and the preprocessing code for the eye-tracking data can be found here (https://git.science.uu.nl/R.S.Hessels/YOUTH_ET_QC/). We have uploaded the Gap-Overlap and questionnaire pre-processing scripts and statistical results on the OSF-platform (https://osf.io/qma6w/).

### Age-related changes in attentional disengagement

We examined the developmental trajectory of attentional disengagement by analyzing the relationship between age and our key measures using Spearman’s rank correlations, which offers straightforward interpretation at the participant-level. Due to our mixed longitudinal and cross-sectional design, we first employed a bootstrapping approach with 5000 randomly generated datasets to construct balanced datasets while maximizing inclusion of unique participants across the full range and avoiding biases from selecting only first measurements. Thus, for participants with multiple measurements, only one measurement was randomly selected per iteration. Importantly, for this part of the analysis, we adopted the actual age of the participants in months instead of the group-categorization.

**Fig. 1:**
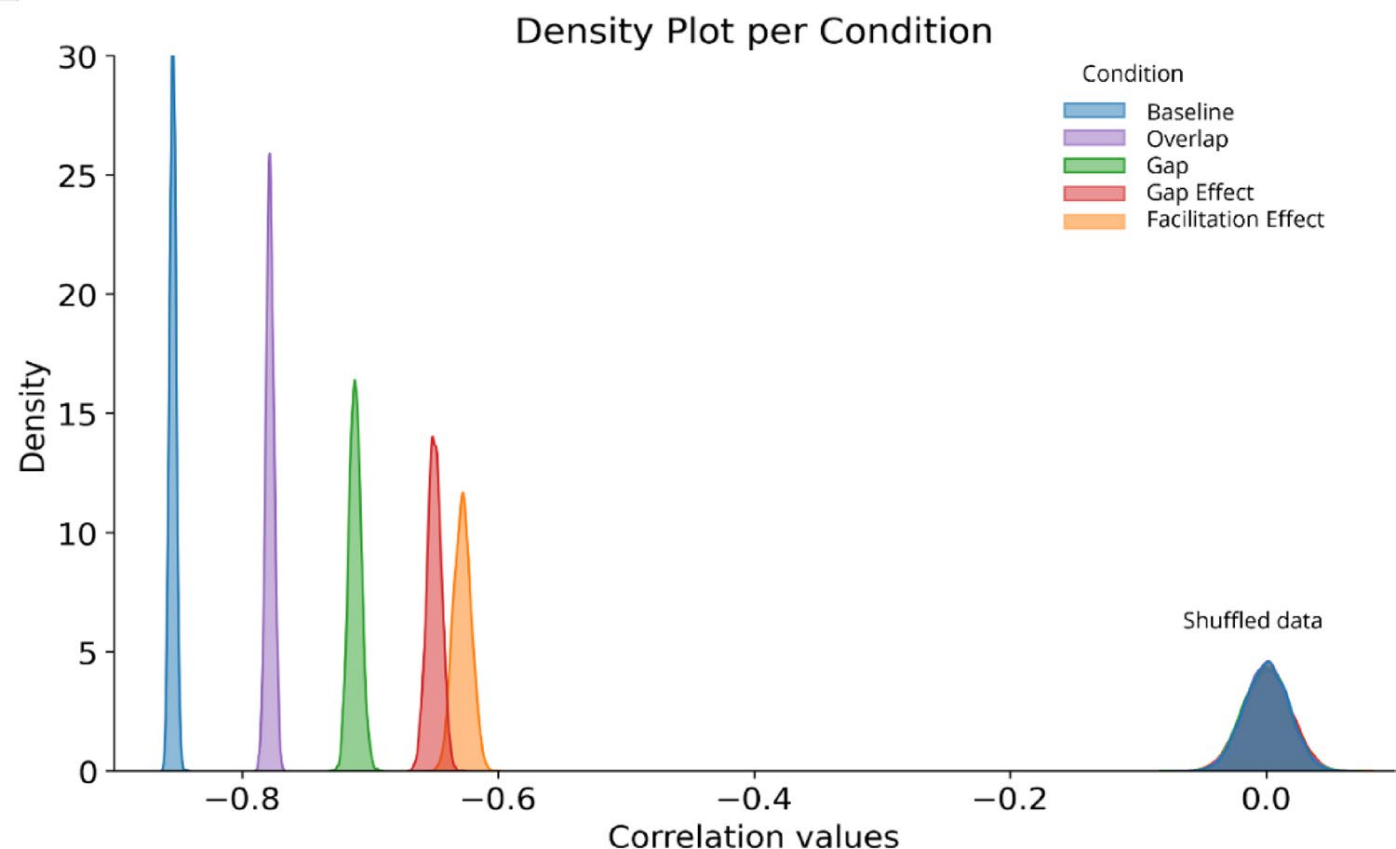
Density plot of the 5000 iteration bootstrapping correlational approach per condition

**Fig. 2:**
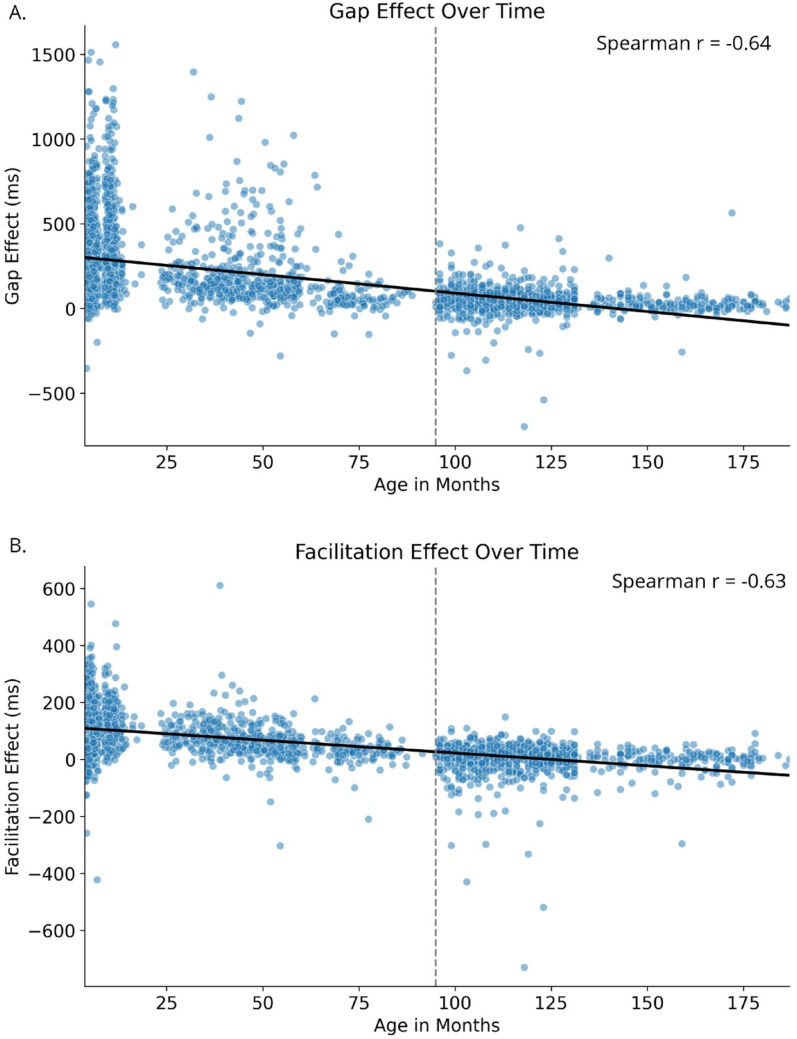
Example scatterplot of a randomly sampled dataset from the bootstrapping approach to illustrate the correlation between age in months and SRTs of the Gap Effect (**A**) and the Facilitation Effect (**B**). Dashed line indicates the border between the Baby Cohort (left) and the Teenage Cohort (right). Note that the linear fit (black line) somewhat underestimates the gap and facilitation effect at later ages due to the non-linear decrease in these measures as a function of age

As visualized in the density plots of all bootstrapping approaches (Fig. 1), our primary measures of interest yielded negative correlations with age, with both the gap effect (median *r* = − 0.650, 95% confidence interval (CI) = [− 0.660, − 0.638], *p* ≤ 0.001) and the facilitation effect (median *r* = − 0.628, *CI* = [− 0.642, − 0.614], *p* ≤ 0.001) decreasing with age (Fig. 2A and B; example of the correlations for one random sample). To validate these findings, we conducted parallel analyses with shuffled age data, which showed no meaningful correlations (gap effect: median *r* = 0.00, *CI* = [− 0.037, 0.037]; facilitation-effect: *r* = 0.00, *CI* = [− 0.035, 0.035]). Additionally, we fitted linear mixed-effects models (LMM) to examine the relationship between age in months (as fixed effect) and both gap effect and facilitation effect including the data for each timepoint, thereby allowing multiple summary measures for participants who have longitudinal data. Random intercepts were included per participant. The LMM showed that both the gap effect and the facilitation effect both significantly declined with increasing age (gap effect: *b* = − 2.257, *p* < 0.001, *z* = − 38.728, *SE* = 0.058, [− 2.372, – 2.143]; facilitation effect: *b* = − 0.896, *p* < 0.001, *z* = − 49.842, *SE* = 0.018, [− 0.931, – 0.861]). These results confirm our patterns observed in our bootstrapping approach, which handles the data set as being cross-sectionally. Subsequent analysis of the individual task conditions also revealed consistent age-related improvements in SRTs. The strongest age-related decrease was observed in the baseline SRTs (median *r* = − 0.854, *CI* = [− 0.859, − 0.849], *p* ≤ 0.001 (shuffled: *r* = 0.00, *CI* = [− 0.034, 0.033]), followed by the overlap conditions SRTs (median *r* = − 0.778, *CI* = [− 0.785, − 0.772], *p* < .001 (shuffled: *r* = 0.00, *CI* = [− 0.034, 0.034]) and Gap conditions SRTs (median *r* = − 0.713, *CI* = [− 0.722, − 0.703], *p* ≤ 0.001 (shuffled: median *r* = 0.00, *CI* = [− 0.036, 0.035]). Shuffled control analysis for all conditions showed no correlations (all *r*’s = 0.00), and all comparisons between shuffled and non-shuffled distributions were significant (all *p*’s < 0.001).

### Longitudinal changes in attentional disengagement

Complementing our cross-sectional correlational approach with within-subject developmental trajectories, we leveraged our dataset’s longitudinal characteristics to examine whether early performance predicted later attentional disengagement abilities. Since the dataset consisted of two independent cohorts, we assessed both separately after having assessed that the Baby Cohort SRTs were significantly longer for all conditions (all *p*’s < 0.001).

In the Baby cohort, we entered the different SRTs (gap effect, facilitation-effect, Baseline, Gap, Overlap) into a repeated measures ANOVA with within subjects’ factor Age (5 months, 10 months, 3 years, 6 years) as this is well-suited for analyzing difference scores. In case of sphericity violations, Huynh-Feldt corrections were applied. To maximize data utilization, we supplemented these analyses and the subsequent planned comparisons with pairwise comparisons (or the non-parametric Wilcoxon’s signed rank test when necessary), allowing inclusion of participants with incomplete visits (see Supplementary for an extensive overview). The overall patterns of results did not qualitatively change, with some time bin comparisons reaching significance with higher power. The Teenage cohort had two measurement points, analyzed using paired comparisons (using paired samples *T*-test or Wilcoxon signed-rank when necessary).

**Fig. 3:**
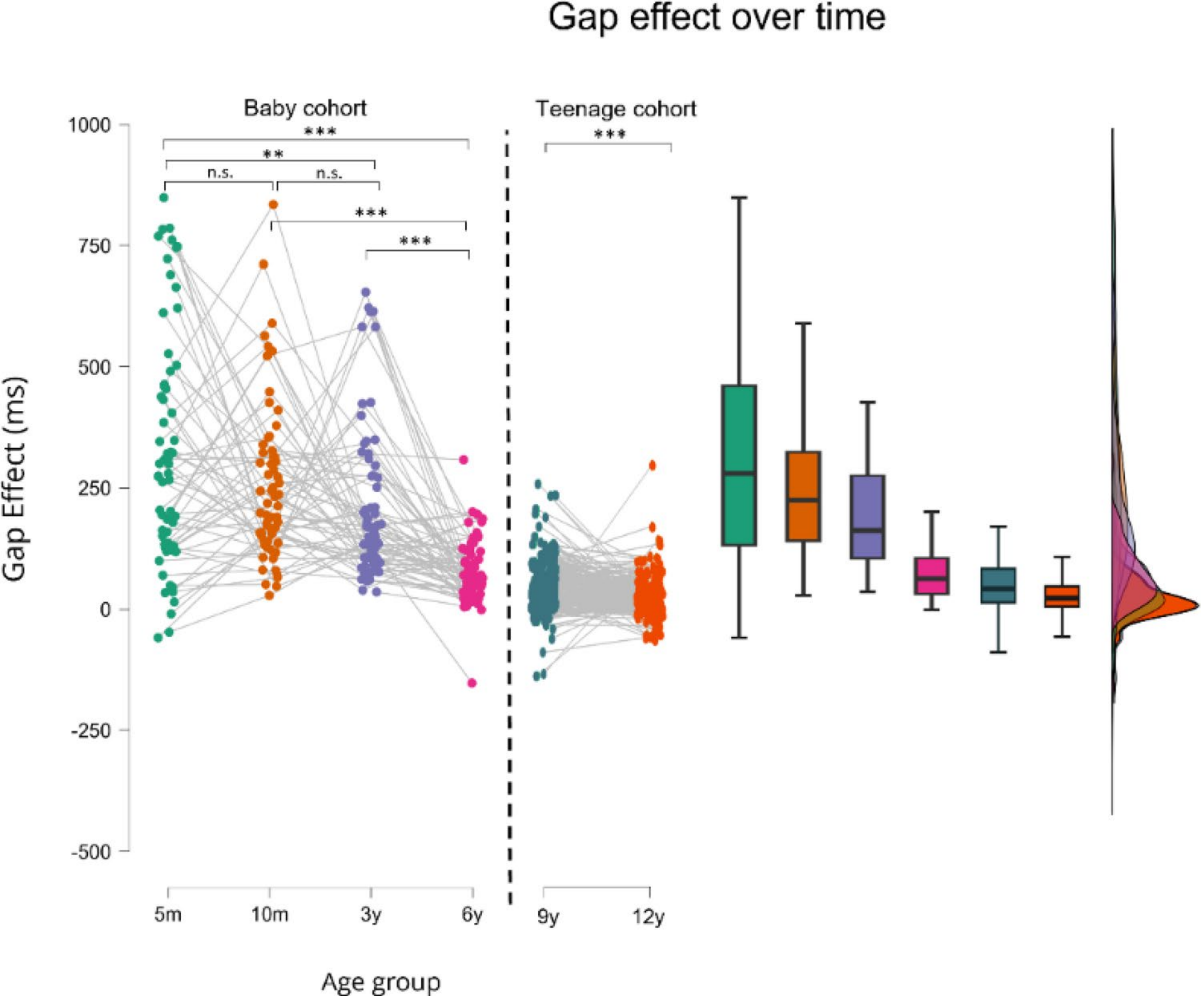
Gap effect (ms) over time for the different age-groups. Dashed line indicates the separation between the baby cohort and the teenage cohort. The gap effect significantly decreased with increasing age. *p < .05, **p < .01, ***p < .001.

*Gap effect*. The gap effect showed significant age-related decreases in both cohorts (Baby Cohort: *F*(2.559, 179.438) = 24.843, *p* < 0.001, *n*^2^*p* = 0.270 ; Teenage cohort: *W*(200) = 13864, *p* < 0.001, *rB* = 0.380). In the Baby cohort, post hoc comparisons revealed no significant changes between the earliest measurements (5 vs. 10 months: *t*(68) = 1.803, *pholm* = 0.102; 10 m months vs. 3 years: *t*(68) = 1.989, *pholm* = 0.102), but significant differences emerged at later ages (all adjusted *p*’s < 0.05, *Cohen’s d* range: 0.667–1.337) (Fig. 3)^1^.

**Fig. 4:**
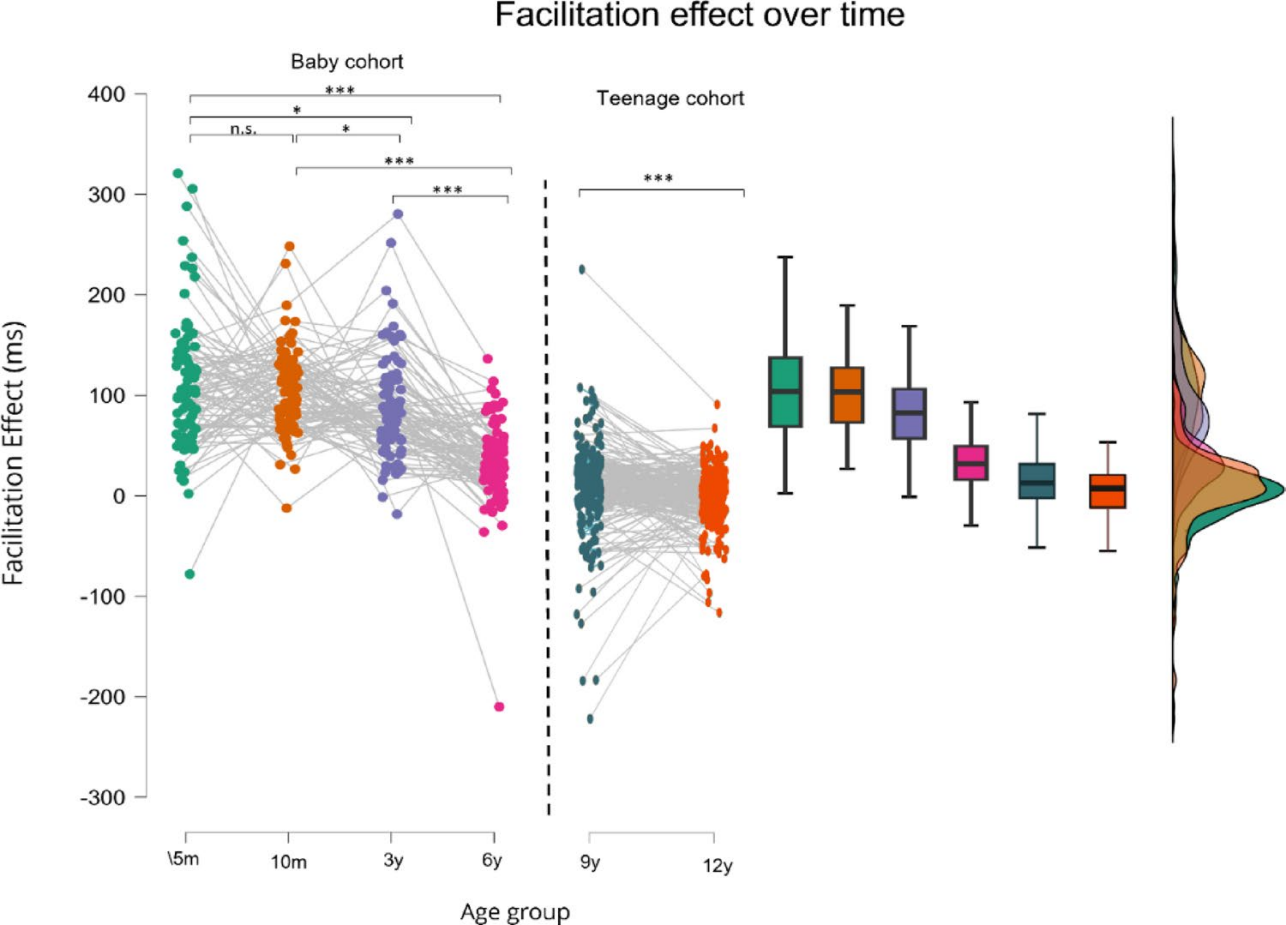
Facilitation effect (ms) over time for the different age-groups. Facilitation significantly decreases over time in the Baby Cohort, but not within the Teenage cohort. Dashed line indicates the separation between the baby cohort and the teenage cohort. *p < .05, **p < .01, ***p < .001.

*Facilitation effect.* The facilitation-effect demonstrated for both the Baby cohort (*F*(2.604, 234.393) = 47.065, *p* < .001, *n*^2^*p* = .343) and the Teenage cohort (*W*(202) = 12921, *p* = .001, *rB* = 0.260) significant age related changes (Fig. 4). Planned pairwise comparisons revealed that the most pronounced differences appeared at age 6 compared to earlier time points (all *t*(91)’s > 8.608, all *pholms*’s < 0.001, *Cohen’s d* range: 1.071–1.538), followed by age 3 compared to earlier time points (5 months vs. 3 years: *t*(91) = 2.680, *p* = .026, 10 months vs. 3 years: *t*(91) = 2.572, *p* = .026) without subsequent differences between 5 versus 10 months (*t*(91) = 0.745, *pholm* = 0.458).

*Task specific measures*. All basic task parameters showed significant developmental changes. In the Overlap condition, SRTs decreased with age in both cohorts (Baby: *F*(2.545, 203.563) = 51.236, *p* < .001, *n*^2^*p* = .390; Teenage Cohort: *W*(330) = 39834, *p* < .001, *rB* = 0.459) with all post-hoc comparisons revealing significance (all *p*’s < 0.005, *Cohen’s d* range: [0.461–1.811]) (Fig. S1). The Baseline condition showed similar age related improvements (Baby Cohort: *F*(2.243,249.027) = 239.989 *p* < .001, *n*^2^*p* = .684; Teenage Cohort: *W*(347) = 44966, *p* < .001, *rB* = 0.489) with all pairwise comparisons being statistically significant (all *p’s* < 0.001, *Cohen’s d* range: [0.639–3.301]) (Fig. S2). The Gap condition also showed significant developmental changes (Baby Cohort: *F*(2.494, 241.926) = 159.875, *p* < .001, *n*^2^*p* = .622; Teenage cohort: *W*(202) = 11994, *p* = .036, *rB* = 0.170) with all comparisons significant (all *p’s* < 0.001, *Cohen’s d* range: [0.650–2.926] (Fig. S3).

### Predictive properties gap effect

Having established that the gap effect decreases over age on a group level, we next set out to examine whether the properties of the gap effect at a younger age exhibited predictive properties over the developmental trajectory. Therefore, we conducted Spearman Rank Correlations between the gap effect at different age points for both the Baby Cohort and the Teenage Cohort to assess this predictive relationship. Our results revealed the strongest correlation between 5 months and 10 months of age, with progressively smaller correlations for larger intervals between measurements (e.g., 5 months−3 years, 5 months−6 years, respectively) (Table 2). Further, the low correlational value in the Teenage Cohort (*r* = 0.04, *p* = .61) aligns with the correlations found across the longer intervals in the Baby Cohort, suggesting that predictive associations are present primarily over shorter developmental windows.

When interpreting these findings, the test-retest reliability of the paradigm needs to be taken into consideration, which has been established at *r* = 0.50 after a one-week interval in 10-month-old infants for a very similar version of the Gap-Overlap task; namely a large pilot study for the YOUth cohort (Cousijn et al. 2017). Given the test-retest reliability of *r* = 0.5 at 10 months, which can be seen as an upper bound estimate, we interpret the small but significant correlation between 5 and 10 months of *r* = 0.18 (*p* < .001) to indicate modest stability in the gap effect across infancy. However, the gap effect at 5 months was not predictive of the gap effect at 3 years or 6 years, indicating that early individual differences in disengagement are not strongly preserved over longer developmental periods.

Although other test-retest reliability values are not available for the older age groups, due to overall better cooperation of the children in eye-tracking experiments at later ages and the higher data quality generally observed, there is good reason to assume that test-retest reliability is higher in later childhood, than in infancy. Yet, despite these favorable conditions, we did not observe significant correlations in the older cohorts. In this regard, our results suggest at least some stability in the gap effect over time at younger ages, whereas these inter-individual differences do not remain across later stages in the developmental timespan. Thus, the limited longer-term predictive value of the early gap effect for later attentional development argues for varying developmental trajectories of attentional disengagement.

**Table 2:** Spearman rank correlation matrix of the gap effect of the baby cohort for all different age groups and the teenage cohort

	10 months	3 years	6 years	9 years
5 months	*r =* .18, *p* < .001	*r =* .04, *p =* .30	*r =* − .01, *p =* .92	–
10 months	–	*r =* .10, *p =* .01	*r <* .01, *p =* .99	–
3 years	–	–	*r =* .04, *p =* .63	–
12 years	–	–	*-*	*r =* .04, *p =* .61

### Correlations between the gap effect and phenotypic attention-related problems

**Fig. 5:**
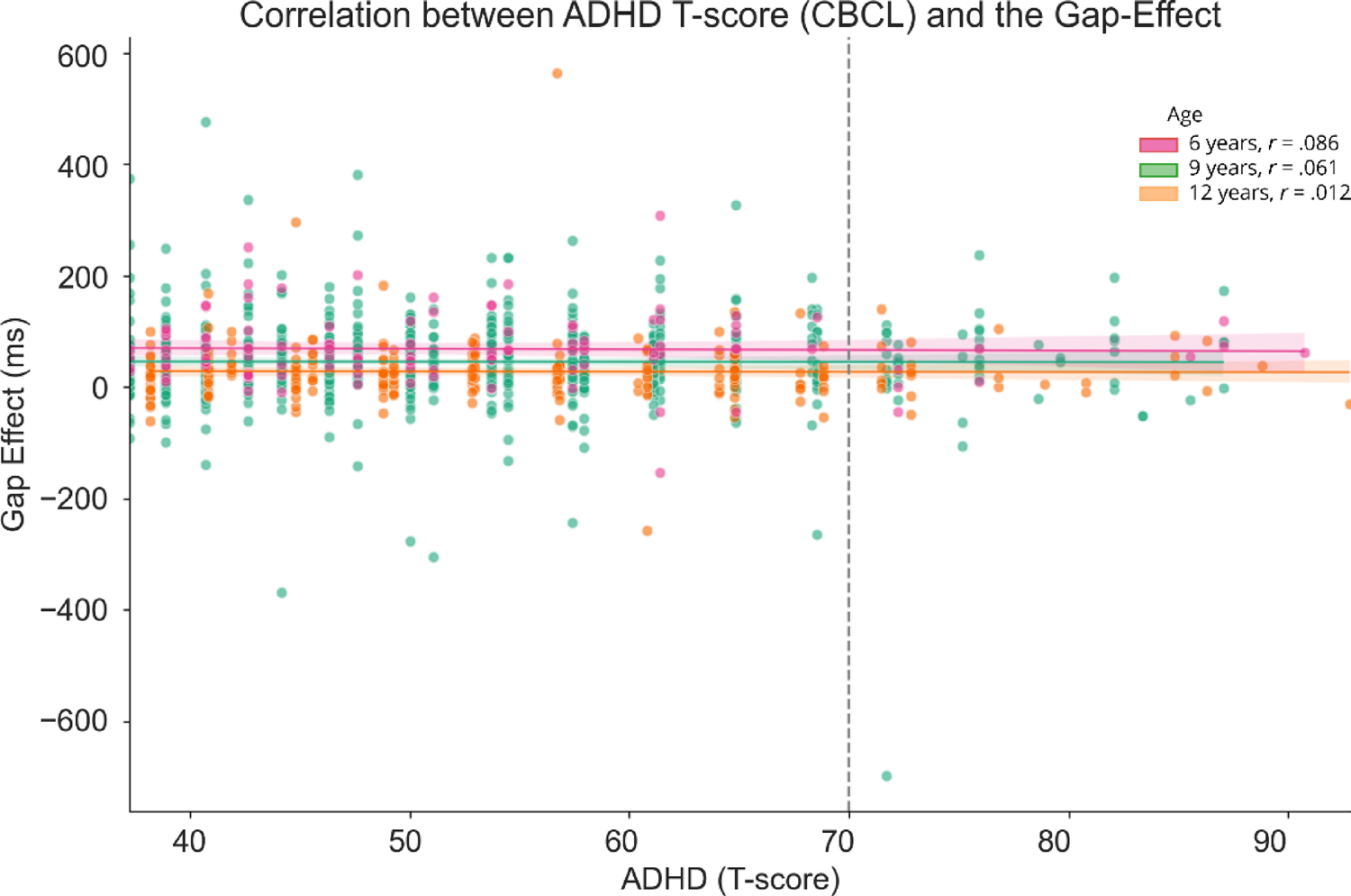
Correlation coefficient between the gap effect (ms) and the ADHD compound score of the CBCL per age group. T-scores are calculated based on norm values. The dashed line indicates the border between the non-clinical range (left) and the clinical range (right)

Lastly, we leveraged the available questionnaire data provided by YOUth to exploratively assess the relation between the gap effect of the Gap-Overlap task and the phenotypic attentional profiles of the children. With the heterogeneous nature of ADHD, we chose to utilize questionnaire scores as continuous variables instead of categorical classifications. This approach allows us to capture the nuanced variations in individual attentional profiles more accurately. Given the exploratory nature of this analysis, we proceeded with separate basic correlation analyses despite the mixed longitudinal and cross-sectional design. By means of a separate Spearman Rank correlations, we examined the relation between the gap effect and several attention-related subscales (converted to t-scores when possible) per age point. Our results revealed no relation between the gap effect and any of the attention related questionnaire subscales of the (E)CBQ and the CBCL (*n* = 99–734 depending on age and measures) (all *r*’s < 0.16, all *p*’s > 0.05) (Supplementary Table 3). To illustrate one example, Fig. 5 visualizes the correlational analysis with *r* = 0.086 for the 6 year olds (n = 113), *r* = 0.061 for the 9 year olds (n = 721) and *r* = 0.012 for the 12 year olds (n = 243) (all *p*’s > .104) for the T-scores of the ADHD subscale of the CBCL. As shown by the dashed line that indicates the separation between considered nonclinical or clinical, only a small group (n = 126, of which 119 unique) fall within the clinical range on *this specific compound* score. For completeness, we repeated the correlation analysis using overall SRT measures from each experimental condition (Baseline, Gap, Overlap), but again found no meaningful relationships with the attention-related questionnaire subscales. These results are not further reported.

## Discussion

To flexibly disengage and shift attention represents a fundamental milestone in cognitive development, enabling infants to effectively explore and learn from their environment (Moyano et al. 2023). Our study provides insights into how these crucial abilities develop, leveraging a robust longitudinal dataset to track the maturation of attentional control across infancy. By examining both between-group differences and within-individual changes in the Gap-Overlap task, we corroborated previously documented developmental patterns while extending the field by revealing important nuances in how attentional shifting abilities unfold over time. Specifically, our findings demonstrate consistent age-related improvements in SRTs at the group level, while simultaneously highlighting substantial individual variation in developmental trajectories. Finally, we found no association between saccadic performance on the Gap-Overlap task and ADHD-related behaviors as measured by the CBCL and the CBQ.

Building on previous research, our study makes several novel contributions to understanding the development of attentional disengagement. While earlier studies have been limited by small sample sizes and restricted age ranges (e.g., Nakagawa and Sukigara 2019; Moyano et al. 2023), our study leverages data from over 3500 participants and extends measurements beyond early infancy into childhood. Our results demonstrate a consistent decrease in SRTs across all task conditions (gap, overlap, and baseline), replicating and extending previous findings while challenging others.

Based on the saccadic reaction times across conditions, our high powered study showed a clear developmental progression towards faster attentional processing, contrasting with Nakagawa and Sukigara (2019) surprising observation that 6-month-olds were faster to disengage than 12-month olds and their later findings suggesting no saccadic differences between 6 and 24 months of age (Nakagawa and Sukigara 2022; Nakagawa et al. 2025). Instead, our results align with studies reporting progressive improvement in early infancy (Alahyane et al. 2016; Gredebäck et al. 2006; Moyano et al. 2023), providing evidence for continuous development of attentional mechanisms or oculomotor control during this critical period.

Regarding development beyond infancy, our extended age range reveals that attentional processing continues to improve well beyond the second year of life. While Moyano et al. (2023) reported a developmental plateau between 9 and 18 months, our measurements at 5 and 10 months, coupled with later childhood assessments, indicate a more protracted developmental timeline. This observation aligns with Nakagawa and Sukigara (2013) finding of shorter disengagement latencies in 36-month-olds compared to younger children, and our data of the later ages further demonstrate that this development continues into early childhood. These findings corroborate observations by Klein (2001) and Klein and Foerster (2001) in children from age 6 onward, as well as Van der Stigchel et al. (2017) who reported shorter SRTs for 12 year-olds compared to 9-year-olds.

Studies reporting no correlation between age and gap and overlap SRTs in children aged 7–12 years (Goepel et al. 2011) or no SRT difference between 10-year-olds and older age groups (11–12 and 13–15 years; Klein et al. 2003) appear contradictory to our findings within the Teenage Cohort. These discrepancies may be attributed to methodological differences, particularly statistical power limitations in previous work. Additionally, Klein et al. (2003) averaged gap and overlap trials in their analysis, potentially masking condition specific developmental patterns.

While examining performance in individual conditions provides valuable insights, the gap effect - the difference in SRTs between overlap and gap conditions - offers a more specific index of attentional control mechanisms. This latency difference reflects distinct attentional states: when attention remains engaged at the fixation point during target appearance (overlap condition), active disengagement is required, whereas pre-emptive disengagement (gap condition) facilitates faster responses (Fischer and Weber 1993). The magnitude of this effect quantifies attentional flexibility and disengagement efficiency, providing a window into developmental processes not captured by examining individual conditions alone. Yet, despite its theoretical importance, remarkably few studies have explicitly examined how the gap effect itself changes across different age groups. To our knowledge, ours is the first longitudinal study to systematically track the gap effect across development in the same individuals over time.

Our analysis moves beyond raw SRT data to disentangle specific attentional mechanisms through difference scores (gap effect and facilitation-effect), allowing us to investigate how these components develop over time while accounting for baseline processing speed. Regarding the emergence of the gap effect, we found no significant differences between children at 5 months compared to 10 months or between 10 months and 3 years. Atkinson et al. (1992) previously identified that the onset of attentional disengagement emerges around the third month when comparing 1-month and 3-month-old infants. Our data extends this work by suggesting that after this initial emergence, disengagement abilities may continue to face developmental challenges throughout infancy, as evidenced by the lack of significant improvements in the gap effect during early developmental periods. Similar results were observed for the facilitation effect between 5 and 10 months. These findings reveal an important developmental dissociation: while basic attentional mechanisms and processing speed improves from early infancy (as shown in our raw SRT data), the development of disengagement abilities follows a slower maturation trajectory than attentional anticipation.

The protracted course of disengagement maturation continues well into adolescence, as evidenced by the negative correlation between the gap effect and age. This finding partially aligns with the few cross-sectional studies examining this phenomenon, such as Van der Stigchel et al. (2017) (9, 12, 15 years) and Klein and Foerster (2001) (6–7, 10–11, 18–26 years), though Eenshuistra et al. (2007) reported no correlation between the gap effect and age (8–9 years vs. 11–13 years vs. young adults). Also, our observations regarding continued differences in the facilitation-effect in the Teenage cohort contrast with Van der Stigchel et al. (2017) finding of no age-related decrease (9, 12, 15 years). These discrepancies highlight the complex nature of this developmental trajectory and the inherent limitations of cross-sectional designs, which may fail to capture developmental nuances due to methodological constraints. For example, cross-sectional studies focusing on developmental stages further into adolescence or adulthood may produce ceiling effects that mask developmental patterns present at younger ages. The substantial differences in oculomotor system development between early childhood and later stages (Johnson 1990) further complicate cross-sectional comparisons across wide age ranges.

Furthermore, to investigate whether individual differences in attentional disengagement remain stable across development, we correlated the gap effect across the different age groups. However, it is important to first acknowledge the methodological constraints that affect our interpretation of these correlations. Experimental paradigms studying individual differences often have limited test-retest reliability (Hedge et al. 2018). Previous research indicates that the test-retest reliability of the gap effect in 10 month old infants is *r* = .5 (Cousijn et al. 2017), which we considered the maximum correlation we could potentially observe across the age groups, with even lower reliability expected for 5 month-old infants due to worse overall eye-tracking data quality (Hessels and Hooge 2019). Given these methodological constraints, our correlation analyses revealed a specific pattern: small but significant correlations were observed within shorter developmental windows (5–10 months, 10 months to 3 years), indicating some individual stability in the gap effect over these periods. However, correlations were absent across longer intervals (5 months to 3 and 6 years, 3–6 years and 9–12 years) suggesting that predictive associations are primarily present within narrower developmental timeframes rather than across multi-year spans. While these findings could be interpreted as evidence for substantial individual variation in developmental trajectories, rather than uniform developmental patterns, this interpretation must be considered alongside the methodological limitations discussed above. The decreased variability and convergence in the gap effect observed in the group-level analysis (indicating increased homogeneity with age) provide additional support for limited long-term predictive value of early measurements. Nevertheless, even with two time points, trajectory information can still provide valuable insights into developmental processes. Future research employing more frequent measurements and improved methodological approaches would strengthen conclusions about individual developmental pathways in attentional control maturation. In sum, our findings align with Moyano et al. (2023) observations of individual differences in developmental trajectories and highlights the complex, non-linear nature of attentional control maturation that cannot be simply extrapolated from early performance.

Given the potentially cascading effects of visual processing on development (see e.g., Keehn et al. (2013), in the context of Autism Spectrum Disorder), we explored whether performance on the Gap-Overlap task corresponded to attentional profiles measured through questionnaires, potentially offering a tool for early recognition of ADHD-related behaviors. Our analyses revealed no significant associations between Gap-Overlap task performance and attention-related subscales on either the CBQ or CBCL. First, it should be noted that ADHD is the extreme of a highly heterogeneous continuum with changing diagnostic criteria over the years. In current design, we correlated specific attentional-related subscales of the questionnaires, thereby ignoring more general attentional profiles within children. Presumably, this limited the predictive value of the parent-reported traits and the gap effect. Furthermore, an important limitation is that while previous studies examined children with established ADHD diagnoses, our sample lacked diagnostic information. We were limited to correlational analyses of parent-reported ADHD-related *traits* within a presumably mostly non-clinical or subclinical population rather than conducting group comparisons with clinically diagnosed individuals. This likely restricted symptom severity potentially explains the absence of significant associations. Our results align with McLaughlin et al. (2021) who similarly found no correlation between parent-reported elevated attentional difficulties (CBCL) and the gap effect.

The literature on ADHD and visual attention paradigms presents mixed findings. While Rommelse et al. (2008) established that ADHD is robustly associated with longer SRTs in visual attention paradigms (e.g., Van der Stigchel et al. 2007; Mason et al. 2003; van der Meere and Sergeant 1988), evidence specifically regarding the Gap-Overlap task remain inconclusive, with conflicting results on saccade latency differences (e.g., Bellato et al. 2023; Maron et al. 2021; Huang and Chan 2020). We exploratively investigated the effects of the more general SRT measures of the task as well, in which we neither found strong correlations between the parent-reported ADHD traits and the more general latencies (SRTs baseline condition, gap condition and overlap condition). The discrepancies in reported findings may stem from various factors, including age-differences between participants and the thereto relating developmental trajectories within the sample, changing diagnostic criteria, the psychometric strength of parent-reported questionnaires and the heterogeneity of ADHD.

Future research should explore additional behavioral measures beyond the gap effect. For instance, examining premature saccades or erroneous saccades might reveal different relationships with attention-related behaviors. Whereas we did not replicate these findings (Supplementary Table S3), some evidence suggests that even within neurotypical populations, slower overall reaction times correlate with higher parent-reported ADHD symptoms. Wall et al. (2024) and Nakagawa et al. (2025) reported a link between longer SRTs in the overlap condition and higher scores on the CBQ Effortful Control subscale.

In conclusion, our study leveraged an exceptionally large dataset spanning multiple developmental periods to provide a comprehensive view of attentional disengagement through both cross-sectional and longitudinal analyses. While we replicated established group-level developmental patterns showing progressive improvement in SRTs, our findings revealed individual variation in developmental trajectories, with early performance showing limited predictive value for later outcomes. This suggests that attentional disengagement undergoes considerable inter-individual change during development, though further research using trajectory-based analyses would be needed to definitively establish distinct developmental pathways. Although we found no associations between Gap-Overlap performance and (parent-reported) attention-related behaviors, this may reflect the difficulty of identifying early markers in highly heterogeneous neurodevelopmental disorders and could be limited by examining subclinical traits rather than diagnosed conditions. The increased understanding of individual developmental patterns established here is a prerequisite for the identification of atypical patterns in clinical assessment. Future studies could build on this work by investigating atypical attention profiles across development and their relationship to Gap-Overlap task performance, particularly in clinical populations where attentional differences may be more pronounced.

## Supplementary Information

Below is the link to the electronic supplementary material.

Supplementary Material 1

## Data Availability

Raw data can be requested via: https://www.uu.nl/en/research/youth-cohort-study/request-youth-data. Experimental code is available via: [https://github.com/UtrechtUniversity/youth-tasks-matlab-kkc/blob/master/saccade_task.m] (https://github.com/UtrechtUniversity/youth-tasks-matlab-kkc/blob/master/saccade/saccade_task.m). Preprocessing code for the eye-tracking data can be found here: . Analyses scripts to reproduce the results and the preprocessing of the questionnaires are available via the Open Science Framework: https://osf.io/qma6w/.

## Footnotes

1 Additionally, an ANOVA comparing the gap effect between participants who completed 48 versus 60 trials (excluding 12-year-olds as they all completed 48 trials) revealed a main effect of trial number (*F*(1, 4420) = 39.149, *p* < .001, η^2^ = .006) but critically no significant Age × Trials interaction (*F*(4, 4420) = 1.968, *p* = .097, η^2^ = .001). This indicates consistent trial number effects across age groups.
