# Supplementary material for: Development of attentional disengagement in typically developing children and children with elevated levels of attentional deficits
Source: Exp Brain Res. 2025 Oct 21;243(11):230. doi: 10.1007/s00221-025-07177-7 (PMC12540621; doi:10.1007/s00221-025-07177-7)
Supplement: Supplementary file 1 — Supplementary Material 1 [file 221_2025_7177_MOESM1_ESM.docx]

**Supplementary**

| **Table S1.** Overview of outlier removal procedure per age group. Values reflect the number of trials and percentages left *after* the specific cleaning procedure. Here, it becomes apparent that most trials were excluded based on premature (<150 ms) or late (>2000 ms) saccades.  Before outlier removal, participants were excluded based on overlapping ages per time bin (n = 16), and due to too many trials (n = 1) | | | | | | | | | | |
| --- | --- | --- | --- | --- | --- | --- | --- | --- | --- | --- |
| **Data cleaning procedure** | | | **All participants** | **5 months** | **10 months** | **3 years** | **6 years** | **9 years** | **12 years** |  |
| Original number of trials (100% of the trials) | | | 344448 | 104921 | 93330 | 50055 | 16565 | 61168 | 18409 |  |
| Eye-tracking data quality cleaning | Mean RMS across the two eyes of > 2° | | 326414  94.76% | 93782  89.38% | 87896  94.18% | 49116  98.12% | 16346  98.68% | 60899  99.56% | 18375  99.82% |  |
|  | Proportion of data loss averaged over 2 eyes > 0.7 | | 296520  86.09% | 81594  77.77% | 76339  81.79% | 45506  90.91% | 14958  90.30% | 59989  98.07% | 18134  98.51% |  |
| Eye-movement filtering | No eye movement made | | 263429  76.48% | 65819  62.73% | 66178  70.91% | 40416  80.74% | 14178  85.59% | 58887  96.27% | 17951  97.51% |  |
|  | Eye movement towards wrong side | | 240470  69.81% | 57848  55.13% | 61626  66.03% | 38343  76.60% | 12896  77.85% | 53443  87.37% | 16324  88.67% |  |
|  | Trials <150 ms (early) or >2000 ms (late) | Total | 187776  54.52% | 47036  44.83% | 52760  56.53% | 32237  64.40% | 10025  60.52% | 35817  58.56% | 9901  53.78% |  |
|  |  | Gap condition | Early: 20509,  Late: 603 | Early: 1933, Late: 264 | Early: 2019, Late: 113 | Early: 2536, Late: 174 | Early: 1533, Late: 12 | Early: 9472, Late: 36 | Early: 3016, Late: 4 |  |
|  |  | Overlap condition | Early: 10422, Late: 11757 | Early: 1157, Late: 5562 | Early: 1005, Late: 4120 | Early: 771, Late: 1701 | Early: 652, Late: 173 | Early: 4804, Late: 186 | Early: 2033, Late: 15 |  |
|  |  | Baseline condition | Early: 8649, Late: 754 | Early: 1560, Late: 336 | Early: 1448, Late 161 | Early: 725, Late 119 | Early: 487, Late 14 | Early: 3075, Late 43 | Early: 1354, Late: 1 |  |
| Conditions filtering | Remove conditions if< 4 trials remained | | 183447  53.26% | 45460  43.33% | 51604  **55.29%** | 31982  63.89% | 9875  59.61% | 34906  57.07% | 9620  52.26% |  |

| **Table S2.**  Percentage of removed trials per age group per condition when eye movements were made towards the wrong side. Note that these percentages add up to 100%. Eye movements made to the wrong side made up for 6.67% of total trials exclusion (see Figure S1) | | | | |
| --- | --- | --- | --- | --- |
|  | | Condition | | |
|  |  | Gap | Overlap | Baseline |
|  | Total | 38.93 | 30.34 | 30.73 |
| Baby and Child | 5 months | 34.42 | 31.99 | 34.34 |
| Baby and Child | 10 months | 36.91 | 32.27 | 30.82 |
| Baby and Child | 3 years | 37.92 | 30.82 | 31.26 |
| Baby and Child | 6 years | 44.54 | 27.61 | 27.85 |
| Child and Teenager | 9 years | 43.75 | 28.27 | 27.98 |
| Child and Teenager | 12 years | 47.39 | 25.26 | 27.35 |

*Figure S1.* SRTs (ms) in the overlap condition over time for the different age-groups. SRTs significantly decrease with increasing age in both Cohorts. Dashed line indicates the separation between the baby cohort and the teenage cohort.
**p* < .05, ***p* < .01, ****p* < .001


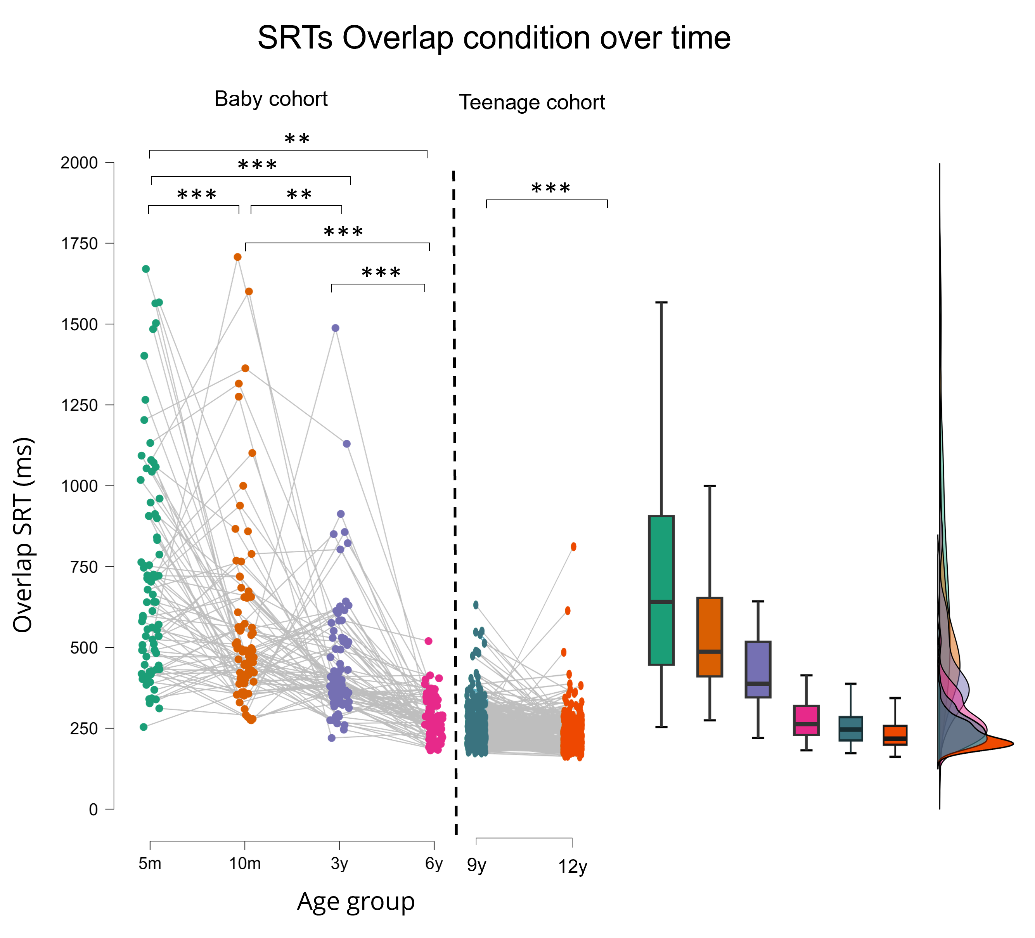


*Figure S2.* SRTs (ms) in the baseline condition over time for the different age-groups. SRTs significantly decrease with increasing age in both Cohorts. Dashed line indicates the separation between the baby cohort and the teenage cohort.
**p* < .05, ***p* < .01, ****p* < .001


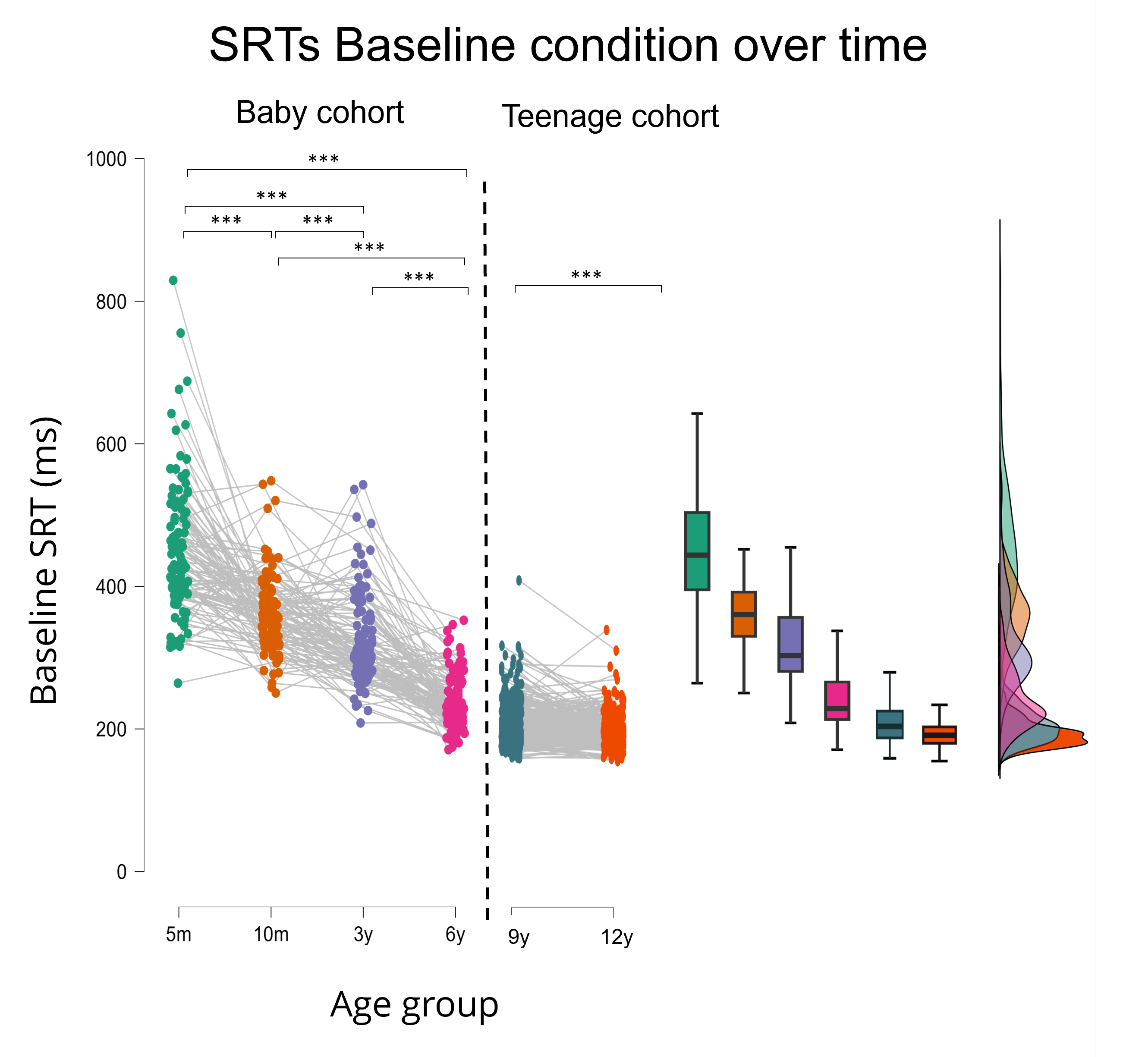


*Figure S3.* SRTs (ms) in the gap condition over time for the different age-groups. SRTs significantly decrease with increasing age in both Cohorts. Dashed line indicates the separation between the baby cohort and the teenage cohort.
**p* < .05, ***p* < .01, ****p* < .001


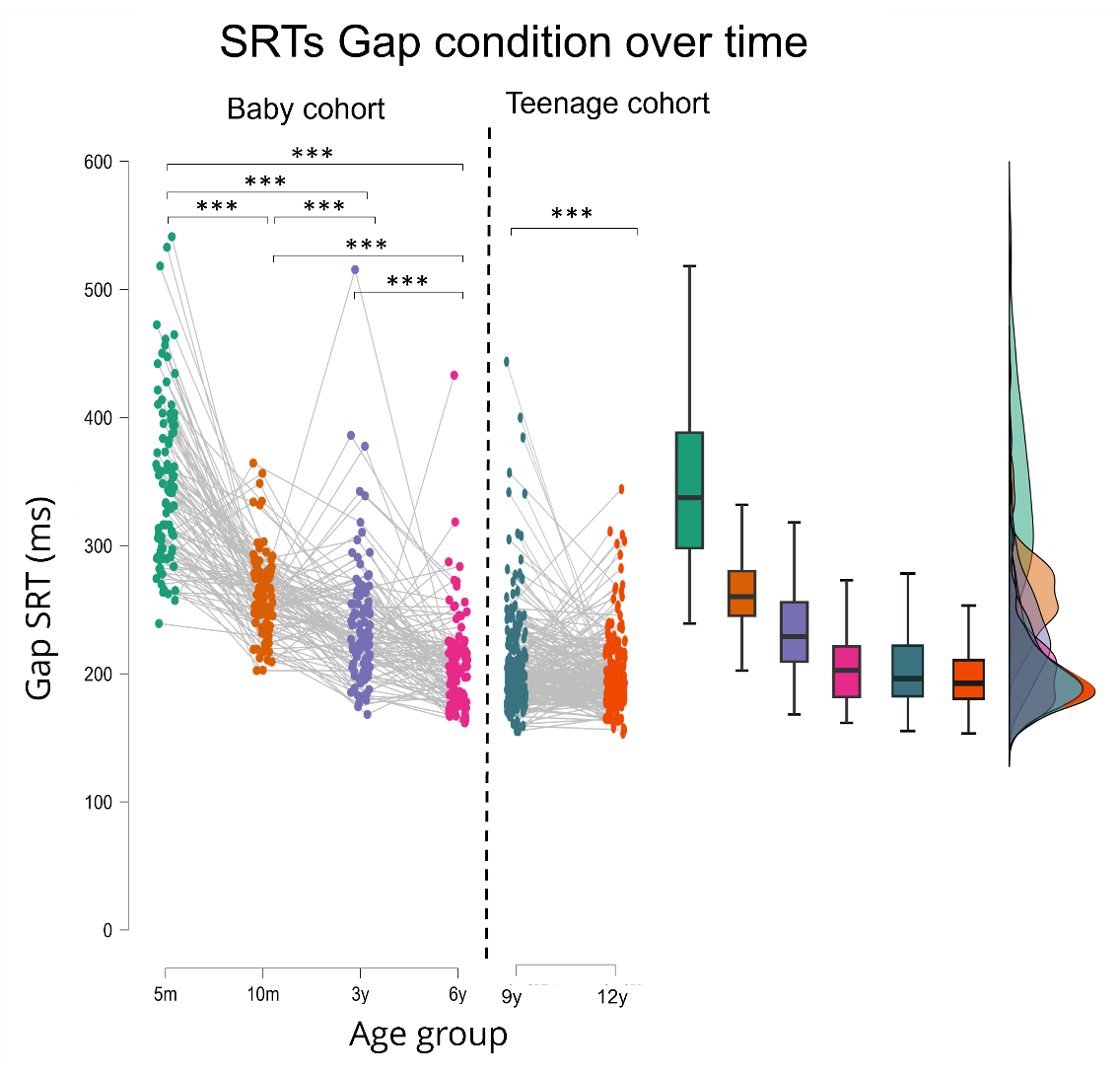


| **Table S3.** Correlational values between the gap effect and attention-related subscales separately for each questionnaire per timepoint. | | | |
| --- | --- | --- | --- |
| **Questionnaire** | **Subscale** | **# participants per wave** | **Correlational measures** |
| ECBQ | Attentional Shifting | 3 years: n = 174 | *r* < .001, *p* = 0.999 |
|  | Attentional Focusing | 3 years: n = 174 | *r* = 0.110, *p* = 0.148 |
| CBQ | Attentional Focusing (T-score) | 3 years: n = 530 | *r* = -.008, *p* = .859 |
|  |  | 6 years: n = 235 | r= .002, p = .977 |
|  | Effortful Control (T-score) | 6 years: n = 235 | *r*= 0.019, *p* = 0.777 |
| CBCL | Attention | 3 years: n = 610 | *r* = .014, *p* = 0.725 |
|  |  | 6 years: n = 99 | *r* = .020, *p* = 0.844 |
|  | Attention (T-score) | 6 years: n = 114 | r = .086, *p* = 0.365 |
|  |  | 9 years: n = 723 | *r* = .068, *p* = 0.066 |
|  |  | 12 years: n = 243 | *r* = .028, *p* = 0.667 |
|  | ADHD | 3 years: n = 610 | *r* = .040, *p* = 0.328 |
|  |  | 6 years: n = 100 | *r* = -.047, *p* = 0.642 |
|  | ADHD (T-score) | 6 years: n = 113 | *r*= .086, *p* = 0.446 |
|  |  | 9 years: n = 721 | *r* = .061, *p* = 0.104 |
|  |  | 12 years: n = 243 | *r* = .012, *p* = 0.858 |
|  | Total T-score | 6 years: n = 116 | *r* = 0.050, *p* = 0.592 |
|  |  | 9 years: n = 734 | *r* = 0.074, *p* = 0.032 |
|  |  | 12 years: n = 245 | *r* = 0.081, *p* = 0.209 |
